# Dr. Jones’s Picnic

**Published:** 1898-05

**Authors:** 


### BOOK NOTICES.

DR. JONES'S PICNIC. By S. E. Chapman, M. D., San Francisco. The Whitaker & Ray Co., Publishers. 1898. Price $1.00.

This is a novel written by a well-known homœopathic physician of California designed to draw public attention to Homœopathy through the medium of a story. This story may be briefly outlined as follows: A benevolent physician of the homœopathic school conceives the idea of making a trip to find the North Pole. He devises a great balloon made of aluminium and inflated with hot air. By means of this air-ship he starts with some pleasant companions to the North pole. On the way he stops in Labrador and becomes the guest of a Canadian hunter whose wife is suffering from cancer. The Doctor prescribes for the sufferer and relieves her of pain, and at the same time brings about recuperation, to the joy of the family who are now filled with hope for the ultimate recovery of the wife and mother.

Then the explorer leaves this abode where he has conferred such a blessing, and ascending into the higher regions of the atmosphere strikes an air current which drives him in an easterly direction until, instead of reaching the North Pole, he finds himself in the interior of Russia in the domain of a great Russian nobleman and close to his castle. The Doctor and his party descend from their air-ship and become the guests of the nobleman. The Doctor finds the Count suffering from sciatic rheumatism and his daughter suffering from consumption. The Doctor again exhibits his homœopathic remedies and cures the Count and relieves the daughter. The Count then detains the Doctor three months at the castle, and during that time the Doctor restores the daughter to health. During this interval the Doctor rescues a Russian princess who is perilously near death from valvular disease of the heart, and restores her to comparative comfort and an endurable invalidism. Then the Doctor leaves his Russian host and finally achieves the object of his desires—the North Pole.

The North Pole, according to this discoverer, seems to be in the centre of a circular island. Upon this island the travelers alight and plant an aluminium pole decorated with an aluminium flag—the stars and stripes—and then they once more enter the car of the balloon and proceed on their journey back to the United States.

This story is certainly very Utopian. There are no difficulties, or at least only such as readily disappear before the slightest effort. There are no dangers, no mistakes, no struggles such as attend ordinary enterprises in this world. The prime object of the author has been to weave a story around a couple of clinical cases treated homœopathically with great success. This object has been attained. The method of taking the case, and of selecting the remedy; the name and character of the drug selected, and the wonderful clinical results attained, are given very accurately and must arrest the attention of all who read the book and show them the really scientific character of a homœopathic prescription and the falsehood of the common order of stories told concerning the system and its methods.

Perhaps the rather Utopian character of the story may beget in the minds of some readers an impression that the cures made by Dr. Jones are also of a Utopian character. They are unconscious of the fact that these cures are but ordinary every-day experiences of those who practice true Homœopathy, and thus the laudable object of the author may be defeated. It is incredibly difficult to make the public believe the actual facts of homœopathic prescribing, familiar though they are to every devoted adherent of the cause.

